# Double half-cone flap umbilicoplasty for proboscoid umbilical hernia in a 2 years old child with satisfactory results 2 years later

**DOI:** 10.11604/pamj.2015.22.44.7818

**Published:** 2015-09-17

**Authors:** Eseme Ebai Ashu, Guifo Marc Leroy, Bang Guy Aristide, Bitang Mafok Louis Joss, Jemea Bonaventure, Savom Eric Patrick, Fotso Guegne Myriam

**Affiliations:** 1 Surgeon Department, University Teaching Hospital Center, Yaoundé, Cameroon; 2 General Surgery and Orthopedic Surgery in the Faculty and Medicine and Biomedical Sciences University of Yaounde 1, Cameroon; 3 Surgeon Departement, Hopital d'Essos (CNPS), Yaoundé, Cameroon; 4 Yaoundé Emergency Center (CURY), Yaoundé, Cameroun; 5 Anesthesiste and Reanimation Department, University Hospital Center, Yaoundé, Cameroon; 6 Gynecology and Obstetrics Department, Polyclinique Innova, Yaoundé, Cameroon

**Keywords:** Double half-cone flap, proboscoid umbilical hernia, umbilicoplasty

## Abstract

Surgical repair of large umbilical hernias may present a challenging surgical problem; standard surgical techniques have proven to be inadequate for both closing the fascial defect of the umbilicus and providing a satisfactory cosmetic result. We describe here a case of double half-cone flap umbilicoplasty that was performed in a 2 years old boy. The case of a 2 years old child with proboscoid umbilical hernia. The protruding umbilical skin was excised sharply by two V-shaped cuts leaving two half cones, a short cephalic (0.5cm) and a long caudal (1cm). A classic herniotomy was carried out, with repair of the facial defect. The caudal half cone was sutured from its apex till half it's length upon itself with interrupted sutures and it was anchored deeply to the fascia. Then we inverted the cephalic half cone which was sutured to the caudal cone to form the new umbilicus. The early result was excellent with no complications and the result after 2years revealed a cosmetically satisfactory shape of the umbilicus. this technique provides a good solution for reconstruction of the protruding umbilical skin and it is easy to learn, easy to be taught and perform in surgical environments and may be applicable for any kind of umbilical reconstruction.

## Introduction

Umbilical hernia is common in children [1]. The management of a large proboscoid umbilical hernia presents challenging problems to the surgeon. As children and parents are concerned with the appearance of the actual hernia, they are also intensely interested in the postsurgical result [1]. The double half-cone flap umbilicoplasty provides a good solution for reconstruction of protruding umbilical skin [2]. In this case, we presented the technique of double half-cone flap umbilicoplasty that allows repair of the facial defect and the management of the redundant skin in such a way as to produce a scarless and naturally appearing umbilicus on an African child in the University hospital center Yaounde Cameroon with satisfactory results after 2 years.

## Patient and observation

**Figure 1:**
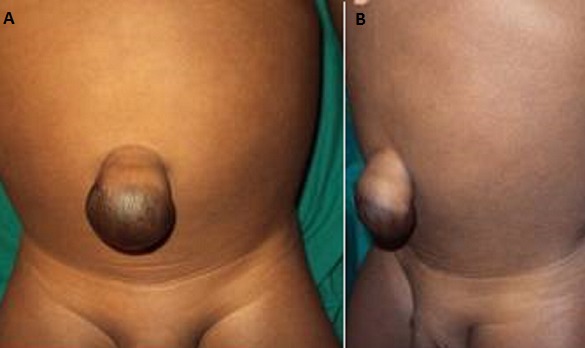
A and B proboscoid hernia

**Figure 2:**
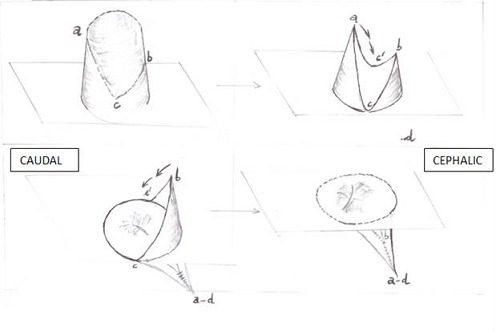
Illustration of the procedure of double half cone umbilicoplasty (ref 2)

**Figure 3:**
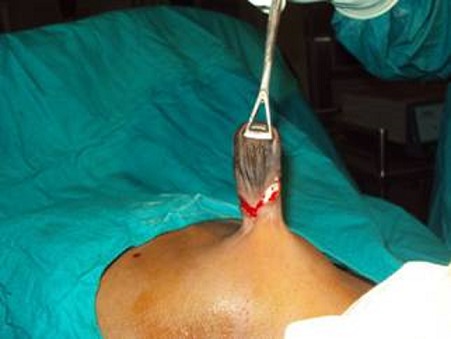
V-shaped cuts leaving two half cones

**Figure 4:**
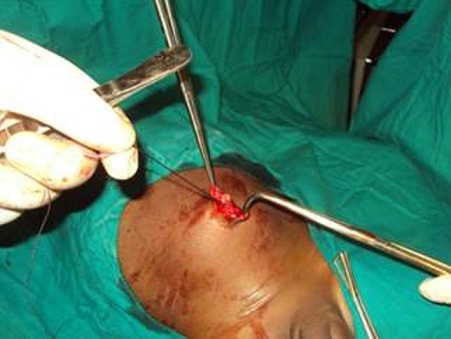
Facial defect repair

**Figure 5:**
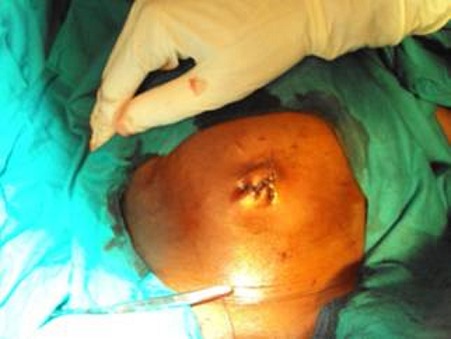
Aspect of the umbilicus at the end of the procedure

**Figure 6:**
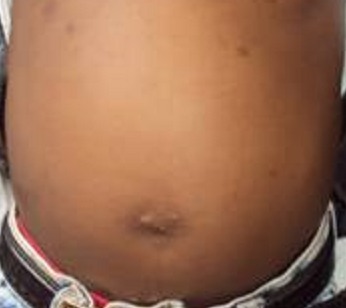
Aspect of the abdominal wall 2 years postoperative

This is a case of a 4 years old child who was brought for consultation in 2013 when he was 2 years old by his parents complaining of persisted unaesthetic umbilical tumefaction from birth with a notion of intermittent pains (Figure 1). On physical examination, the child presented with an umbilical hernia with protruding skin. A surgical indication was the choice of management but the parents remained skeptical with what the esthetical outcome will look like. After consent was obtained from the parents, the technic we opted to use was the double half-cone flap umbilicoplasty. Under general anesthesia the apex of the protuberant umbilical skin is held with a forcep (Figure 2). The protruding umbilical skin was excised sharply by two V-shaped cuts leaving two half cones, a short cephalic (0.5cm)and a long caudal(1cm) (Figure 3). A classic herniotomy was carried out, with repair of the facial defect. The caudal half cone was sutured from its apex till half it's length upon itself with interrupted sutures and it was anchored deeply to the fascia (Figure 4). Then we inverted the cephalic half cone which was sutured to the caudal cone to form the new umbilicus(Figure 5). The final results of the technique 2years postoperatively are satisfactory (Figure 6).

## Discussion

Umbilical hernia is common in children [1]. The normal location of the umbilicus is the superior border of the iliac crest [3]. Umbilical hernia in children is classified according to the diameter of the facial defect: class 1 in which the defect diameter is between 1.5,and class 2 in which the defect is between 1.5 and 3cm and class 3 in which the defect is more than 3cm. More than 95% of class 1 defects will close by age 5 without surgery, while larger defects of classes 2 and 3 seldom close spontaneously [4, 5]; both class 2 and 3 represent the clinical oblique type described by Blumberg, which is a large hernia with downward displacement of the umbilicus and appearing as descending from above(the proboscoid variety) [4]. In another classification, the deformities are classified into 5 types Type 0: the defect of umbilicus; Type I, the low-grade protrusion; Type II, the high-grade protrusion with wide base; Type III, the high-grade protrusion with narrow base; and Type IV, the protrusion in depression. Huge umbilical hernias are voluminous umbilical hernias that are frequent in black African children [6] and the umbilicus is an important aesthetic feature of the abdomen [7]. Incarceration is not as uncommon as thought. In a retrospective study carried out by Chirdan LB and collaborators in 2006 in a series of Fifty-two children with umbilical hernias were seen in the hospital over the period. Twenty-three (44.2%) had incarceration. Seventeen (32.7%) had acute incarceration while 6 (11.5%) had recurrent incarceration. Incarceration occurred in hernias of more than 1.5 cm in diameter in those whose defect size was measured [1]. The anxiety of parents being the redundant umbilical skin following closure of the facial defect rather than the hernia defect itself [2]. Several techniques are used in their treatment for umbilical reconstruction, many techniques which have been described for facial defects repair have limitations with non satisfactory esthetic results. While techniques using skin flaps provide better aesthetic results [6]. The aim of the surgeon is to create an umbilicus of natural appearance, which consists of a ring, a tubular wall, a sulcus, and a bottom without any excess skin to preserve the aesthetic aspect of the umbilicus [8] knowing that the ideal umbilicoplasty creates a permanent rounded depression in the midabdomen with minimum scarring with the upper margin having a slightly hooded skin [8]. The major concern we had with this case, was to show how the repair of facial defect and umbilicoplasty in the same setting with the double half-cone flap umbilicoplasty was a good solution for the reconstruction of protruding umbilical skin [9] and contributes to alleviate both parental worry and child suffering which could be increased if umbilicoplasty is postponed to a separate session in preadolescence [10]. It remains an easy procedure to perform and should be encouraged amongst learners and surgeons.

## Conclusion

This technique of double half-cone flap umbilicoplasty is well-adapted to huge umbilical hernias in children. It is a simple method and assures a satisfactory anatomical and cosmetic result.
